# Blood-based identification of non-responders to anti-TNF therapy in rheumatoid arthritis

**DOI:** 10.1186/s12920-015-0100-6

**Published:** 2015-06-03

**Authors:** Ty M. Thomson, Reynald M. Lescarbeau, David A. Drubin, Daphna Laifenfeld, David de Graaf, David A. Fryburg, Bruce Littman, Renée Deehan, Aaron Van Hooser

**Affiliations:** Selventa, Inc., Cambridge, MA USA; Translational Medicine Associates, Stonington, CT USA

**Keywords:** Rheumatoid arthritis, Infliximab, Anti-TNF therapy, Classifier

## Abstract

**Background:**

Faced with an increasing number of choices for biologic therapies, rheumatologists have a critical need for better tools to inform rheumatoid arthritis (RA) disease management. The ability to identify patients who are unlikely to respond to first-line biologic anti-TNF therapies prior to their treatment would allow these patients to seek alternative therapies, providing faster relief and avoiding complications of disease.

**Methods:**

We identified a gene expression classifier to predict, pre-treatment, which RA patients are unlikely to respond to the anti-TNF infliximab. The classifier was trained and independently evaluated using four published whole blood gene expression data sets, in which RA patients (n = 116 = 44 + 15 + 30 + 27) were treated with infliximab, and their response assessed 14–16 months post treatment according to the European League Against Rheumatism (EULAR) response criteria. For each patient, prior knowledge was used to group gene expression measurements into disease-relevant biological signaling mechanisms that were used as the input features for regularized logistic regression.

**Results:**

The classifier produced a substantial enrichment of non-responders (59 %, given by the cross validated test precision) compared to the full population (27 % non-responders), while identifying nearly a third of non-responders. Given this classifier performance, treatment of predicted non-responders with alternative biologics would decrease their chance of non-response by between a third and a half, substantially improving their odds of effective treatment and stemming further disease progression. The classifier consisted of 18 signaling mechanisms, which together indicated that higher inflammatory signaling mediated by TNF and other cytokines was present pre-treatment in the blood of patients who responded to infliximab treatment. In contrast, non-responders were classified by relatively higher levels of specific metabolic activities in the blood prior to treatment.

**Conclusions:**

We were able to successfully produce a classifier to identify a population of RA patients significantly enriched in anti-TNF non-responders across four different patient cohorts. Additional prospective studies are needed to validate and refine the classifier for clinical use.

**Electronic supplementary material:**

The online version of this article (doi:10.1186/s12920-015-0100-6) contains supplementary material, which is available to authorized users.

## Background

Rheumatoid arthritis (RA) is a chronic and debilitating autoimmune disease that primarily affects synovial joints. Currently, the most prevalent targeted therapies in RA are the anti-tumor necrosis factor (anti-TNF) agents infliximab, adalimumab, and etanercept, which act to neutralize the signaling of this pro-inflammatory cytokine [1–4]. However, a substantial proportion of patients (approximately 30-40 %) fail to respond to anti-TNF therapy, exposing them to unnecessary adverse effects as their disease progressively worsens [1, 4]. Abatacept (T-cell inhibitor), rituximab (anti-CD20; B-cell inhibitor) and tocilizumab (anti-interleukin 6 receptor; anti-IL6R) have all been shown to be efficacious in many patients that fail anti-TNF therapy [5, 6]. Thus, there is a clear need for a test to provide early identification of the subset of patients who are unlikely to respond to anti-TNF therapies so that these patients can be given alternative treatment options to reduce further disease progression.

The overall likelihood of non-response to anti-TNFs and other biologics are similar (around 30-40 % [7]). Thus, if such a test could provide a high confidence prediction that a patient has an elevated likelihood of not responding to anti-TNF therapy, then it would indicate that this patient could have a greater chance of responding to an alternative biologic. For example, if a test were to inform a patient that they only had 40 % chance of responding to anti-TNF therapy, then their best course of action would be to receive an alternative biologic therapy, increasing their chance of responding to therapy from 40 % (on anti-TNF therapy) to 60-70 % (on an alternative biologic).

Although significant efforts have been made to identify classifiers (biomarkers or biomarker panels) to predict response to targeted biologic therapies in RA, predictive value has not generally been observed across independent cohorts. Several studies have presented blood-based gene expression classifiers that appear to predict response to anti-TNF [8–12], anti-IL6 [13] and anti-CD20 [14] therapies. However, not all reported performance measures accurately reflect how the classifiers would be expected to perform on new samples (see [15] for an overview of common problems with gene expression classifiers). Additionally, one study found that of eight reported gene expression signatures for predicting response to anti-TNF therapy, most failed to produce predictive value in an independent test cohort [16], although the statistical significance these findings were not evaluated.

We investigated whether a blood-based gene expression classifier could predict response to a targeted biologic therapy in RA when subjected to statistically rigorous evaluation across multiple patient cohorts. The current study focused on identifying likely non-responders to the anti-TNF treatment infliximab, a chimeric monoclonal antibody, due to availability of suitable gene expression data sets with accompanying response data. To overcome some of the heterogeneity of these different studies, we used novel systems biology methods that integrate multiple simultaneous measures of gene expression with a priori knowledge of thousands of protein and gene regulatory relationships, including immune-related pathways. Multiple gene expression readouts for individual signaling mechanisms produce a quantitative assessment of the mechanisms driving signaling in individual patients from the downstream effects of these mechanisms on gene expression [17].

## Methods

### Data analysis

All data used in this study (Table 1) came from clinical trials that were approved by the appropriate ethics committees, and our analysis of this public data did not require ethical approval. Statistical methods were performed using the R software package version 3.1.3 [18]. Raw data were downloaded from GEO [16] and processed using tools from the *limma* package [19]. Affymetrix CEL files were processed using the brainarray chip definition file version 17.1.0 ENTREZG [20] where possible. Technical replicates were averaged for GSE11827 and GSE3592, with the exception of samples GSM82658 and GSM82661 from GSE3592 which were omitted because they were ambiguously annotated to patient ID. Probes and probe sets were mapped to Entrez Gene IDs, and those that corresponded to multiple Entrez Gene IDs were omitted from further analysis. Where multiple probes or probe sets corresponded to a single Entrez Gene ID, average expression was computed.

**Table 1:** Data sets used in this study

Data set	Use in this study	Microarray platform	Blood sample	Treatment	Co- Therapies	Previous Treatment with Biologics	Response Criteria (Time after Treatment)	Patient breakdown
GSE12051 [10]	Classifier training	Sentrix Human-6 Expression BeadChip	Whole blood	Infliximab	All pts: MTX, prednisone and NSAID	No anti-TNFs	EULAR DAS28 poor: NR	7 NR, 37 R
							EULAR DAS28 moderate/good: R (14 weeks)	
GSE19821 [36]	Classifier training	Stanford Custom Array	Whole blood	Infliximab	All pts: MTX	No anti-TNFs	EULAR DAS28 poor: NR	5 NR, 10 R
					Some pts: prednisone and/or NSAID			
							EULAR DAS28 moderate/good: R (16 weeks)	
GSE58795 [37, 38]	Classifier training	Human RSTA Custom Affymetrix 2.0	Whole blood	Infliximab	All pts: MTX	No biologics in prev. 3 months	EULAR DAS28 poor: NR	7 NR, 23 R
					Some pts: prednisone, DMARDs and/or NSAIDs		EULAR DAS28 moderate/good: R (14 weeks)	
GSE33377 [16]	Classifier training (infliximab) and evaluation (adalimumab)	Affymetrix Human Exon 1.0 ST Array	Whole blood	Infliximab or Adalimumab	All pts: MTX	No anti-TNFs	EULAR DAS28 poor: NR	Infliximab: 13 NR, 14 R
							EULAR DAS28 good: R (14 weeks)	Adalimumab: 11 NR, 4 R
GSE42296 [39]	Classifier evaluation	Affymetrix Human Gene 1.0 ST Array	PBMCs	Infliximab	All pts: MTX	No anti-TNFs in prev. 3 months	ACR0/20: NR	13 NR, 6 R
					Some pts: prednisone and/or NSAID		ACR50/70: R (14 weeks)	
GSE3592 [8]	Classifier evaluation	INSERM Homo sapiens 14 K array_Liverpool2	PBMCs	Infliximab	All pts: MTX and prednisone	Unknown	ΔDAS28 < 1.2: NR	7 NR, 6 R
					Some pts: NSAIDs		ΔDAS28 ≥ 1.2: R (3 months)	
GSE25160 [40]	Classifier evaluation	Affymetrix Human Gene 1.0 ST Array	PBMCs	Tocilizumab	All pts: MTX	No anti-TNFs	ACR0/20: NR	4 NR, 9 R
					Some pts: prednisone		ACR50/70: R (14 weeks)	
GSE37107 [41]	Classifier evaluation	Illumina HumanHT-12 V3.0 Expression BeadChip	Whole blood	Rituximab	Some pts: prednisone, DMARDs and/or NSAIDs	Discontinued anti-TNF, at least 1 month wash out period	ΔDAS28 ≤ 1.2: NR	6 NR, 8 R
							ΔDAS28 > 1.2: R (6 months)	
GSE11827 [42]	Classifier evaluation	INSERM Homo sapiens 14 K array_Liverpool3	PBMCs	Anakinra	All pts: MTX	None	ΔDAS28 < 1.2: NR	7 NR, 7 R
					Some pts: prednisone or NSAID		ΔDAS28 ≥ 1.2: R (3 months)	

*ACR*, American College of Rheumatology; *DAS28*, disease activity score using 28 joint counts; *DMARD*, Disease-modifying antirheumatic drug; *EULAR*, European League Against Rheumatism; *MTX*, methotrexate; *NR*, non-responder; *NSAID*, nonsteroidal anti-inflammatory drug; *PBMCs*, peripheral blood mononuclear cells; *pts*, patients; *R*, responder

### Mechanism strength scoring

Gene expression measurements in each data set were grouped into biological mechanisms for classifier training and evaluation. Specifically, a mechanism is defined as a set of genes that were previously identified as differentially expressed in response to a change in the activity or abundance of a biologically active molecule, as well as the of direction of regulation for each gene in the gene set [21]. Each gene with increased or decreased in expression in a mechanism is based on one or more cause and effect relationships curated from peer-reviewed, published literature [17].

The Strength algorithm provides a measure of the relative activity for any given mechanism based on the differential expression of genes in that mechanism [17]. For the purposes of this study the differential expression of each gene was computed with respect to the population median within each data set. Briefly, the strength score for a mechanism is the weighted mean of the (log2) differential expression of genes, where the weighting factors are the direction of regulation of gene expressions in the mechanism based on prior observation (+1 for increase relationships, −1 for decrease relationships).

As the data sets were collected on different microarray platforms (Table 1), the set of genes used to compute the strength score for a given mechanism differed somewhat for each data set. In total, classifier development was restricted to 1667 mechanisms from the Selventa knowledge base that had at least four genes measured in each of the six infliximab data sets (see Additional file 1 for the list of 1667 mechanisms and their strength scores).

### Classifier development

Multi-mechanism linear models were constructed through regularized logistic regression (lasso regularization) using the *glmnet* R package [22]. Model parameters were rescaled such that the classifier scores fell between 0.5 and 9.5 for the training samples. Classifier scores from test samples that were less than zero were set equal to zero, and scores greater than ten were set equal to ten. Classifier score thresholds were selected such that 60 % of the non-responders in the training cohort fell above the threshold (60 % non-responder sensitivity on the training cohort), a strategy that we found to be effective for identifying a group of non-responders with high specificity in the test cohorts.

Since each training data set was measured on a different microarray platform, there was no expectation that the signal for each data set would be directly comparable. However, when samples are compared against a common reference, expression values between various microarray platforms have an approximate 1:1 ratio [23, 24]. Here we compared each sample to the median sample for that data set under the assumption that the median patient from each data set would be similar. However, the small sample sizes of the data sets and the differences in the ratios of anti-TNF responders to non-responders in each data set suggest that the median samples likely differ somewhat between the studies.

### Classifier validation

Classifier performance was estimated using cross validation. To provide an in-batch estimate of performance, repeated 10-fold cross validation was used (1000 repeats) to reduce the variance of the performance estimates compared to leave-one-sample-out cross validation [25]. For 10-fold cross validation, samples were randomly divided into ten groups, where each of the training data sets was split approximately equally among the groups. A classifier was then trained as described above on samples from nine of the ten groups, and tested on the left out group. The training/test scenario was repeated until each group served as the test group exactly once. The whole process repeated for a total of 1000 cross validation repeats. To provide an out-of-batch estimate of performance, leave-one-batch-out cross validation was performed by training on each combination of all but one data set and testing on the left-out data set. One-sided AUROC p-values were computed using the Wilcox rank sum test.

### Evaluation of previously published classifiers

Five previous studies have described eight different gene expression classifiers for predicting response to anti-TNF therapy in RA from blood. Here we denote each classifier by the study author name and number of genes in the classifier, and indicate the anti-TNF therapy and blood sample type used for classifier training: Lequerré_20 and Lequerré_8 (infliximab treatment, PBMCs) [8]; Julia_8 (infliximab, whole blood) [10]; Stuhlmuller_82, Stuhlmuller_11, and Stuhlmuller_3 (adalimumab, monocytes) [12]; Tanino_8 (infliximab, whole blood) [11]; and Sekiguchi_18 (infliximab, whole blood) [9]. Only one gene from Stuhlmuller_3 was measured across all four whole blood infliximab data sets, and thus we omitted this classifier from our analysis. Because each of these studies only provided a list of genes but no mathematical formula for predicting response from these genes, each classifier was re-trained on the four whole blood data sets using the specified genes and same clustering method specified in the original studies. The performance of each classifier was assessed via leave-one-sample-out cross validation across the four whole blood infliximab data sets. Julia_8 was originally trained on GSE12051, so GSE12051 was omitted from cross validation for Julia_8 to avoid feature selection bias [15]. With the exception of Julia_8, classifier scores were determined based on the distance between the test sample and the median of each cluster, and response predictions were based on the response call assigned to the nearest cluster median. Julia_8 uses the k-nearest neighbors algorithm (k = 3) which does not produce discrete clusters, so predicted non-responders were assigned classifier scores of 1, and predicted responders were assigned scores of 0.

### Classifier error Due to uncertainty in DAS28 scores

Response calls are often based on DAS28 scores, which are known to have a measurement standard deviation of 0.6 [26]. To estimate the maximum expected classifier performance based on this known measurement error, the corresponding measurement noise was imposed onto the DAS28 measurements from GSE58795 (the only data set for which, in addition the response calls, individual DAS28 scores were available), and alternative noisy response calls were determined for each patient (using EULAR DAS28 response calls). The original ΔDAS28 scores were used as the classifier scores, representing a perfect classifier based on the “true” DAS28 scores underlying the noisy response calls. The AUROC was then calculated using this classifier to predict the noisy response calls. The process was repeated 1000 times and the median AUROC was computed to represent the maximum expected performance of a classifier for the prediction of DAS28-based response calls.

## Results

### Datasets for classifier training

Public databases were screened extensively for candidate data sets to be used in development of a blood-based response classifier for infliximab treatment in RA. Four data sets were identified from North American or European cohorts of RA patients that contain transcriptome-wide measurement of gene expression at baseline in whole blood and unambiguously annotated standard clinical response criteria to infliximab therapy: Gene Expression Omnibus (GEO) accession IDs GSE12051, GSE19821, GSE58795, and GSE33377. Although the published annotations for GSE33377 do not identify the patients in this study that received infliximab or adalimumab treatment, the authors of this study kindly provided this information (M. Coenen, personal communication) allowing for the classifier to be trained exclusively on infliximab-treated patients. Whole blood infliximab data sets were the focus of the present study because they provide a sufficient number of total patients (n = 116) for classifier training, and response calls in these studies were all based on the same criteria (Table 1). Clinical measures, such as age, gender, ethnicity, and previous disease treatments, were not available for individual patients across all studies and thus were not used for classifier training or characterization. Patient populations, experimental protocols, gene expression profiling, and response criteria differed somewhat in each study (Table 1).

### Classifier development

Gene expression measurements in each training data set (GSE12051, GSE19821, GSE58795, and GSE33377_infliximab_) were median centered and grouped into 1667 biological mechanisms for classifier training and evaluation (see Methods). The Strength algorithm [17] was used to provide a measure of the relative activity for each mechanism based on the differential expression of genes in that mechanism. Regularized logistic regression on mechanism strength scores and response calls from the four whole blood infliximab data sets produced a classifier model for anti-TNF non-response consisting of a linear combination of 18 mechanisms (Table 2). The classifier threshold was specifically tuned to for higher specificity at the expense of sensitivity in order to provide high confidence identification of non-responders (see Methods).

**Table 2:** Classifier linear model

Mechanism	Coefficient	Number of supporting genes
(Constant)	4.80	-
CDK2	0.57	9-11
DPPA4	4.33	32-49
ERBB2	16.32	146-180
FOXA2	0.84	91-106
Gamma secretase	3.59	81-96
IL11	−4.99	27-32
MAP2K3	1.15	5-6
MBD1	−0.23	5-8
MEIS1	1.40	89-118
MST1R	−0.56	32-39
NF1	−3.02	66-77
NFE2L2	5.22	122-145
Norepinephrine	2.21	28-36
NOS2	−0.12	9-10
NR2F6	−0.85	5-6
PPARG	4.60	496-630
S100A8/S100A9 complex	−4.76	16-19
Sulindac sulfide	−4.13	21-26

Patients with classifier scores above 5.96 are predicted non-responders and thus potential candidates for alternative biologic therapy. Patients with classifier scores below this threshold were not predicted as non-responders and are assumed to be a mix of responders and non-responders. The number of genes supporting each mechanism varies based on the microarray platform used in each of the four training data sets. Note that the magnitudes of the coefficients do not necessarily indicate relative importance of the mechanisms for predicting non-response

### Evaluation of classifier performance

The gold standard for evaluating the performance of a classifier is to test the classifier on a large independent validation cohort. However, given the limited amount of available data on which to train and evaluate the classifier, bias can be inadvertently introduced in the selection of a small validation cohort, such that selection of a different subset of the samples to serve as the validation cohort might significantly affect results. We employ two statistically rigorous approaches to validate the classifier in the face of the limited number of samples.

**Fig. 1:**
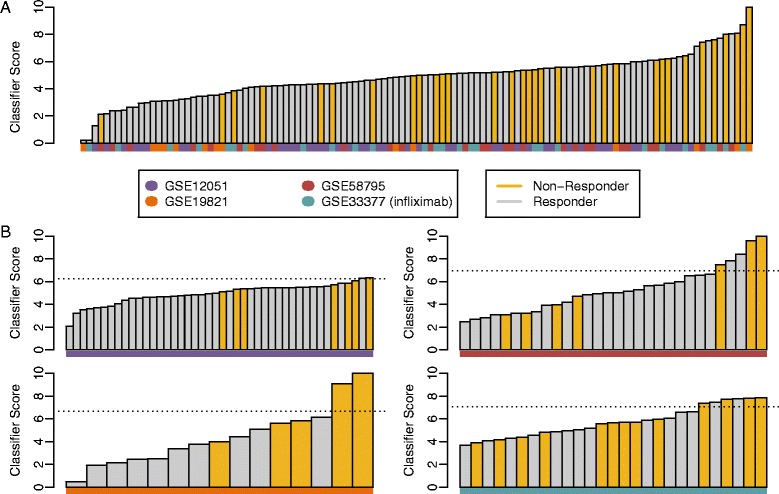
Patient stratification from cross validation. The patient stratification plot shows the whole blood classifier score for each patient for (**a**) a representative 10-fold cross validation matching the median AUROC of 71 % across all cross validation repeats, and (**b**) leave-one-batch-out cross validation. Patients were sorted by classifier score and colored by their clinical response calls. The dotted lines represent the classifier threshold for predicting non-responders. No classifier threshold is shown for 10-fold cross validation because patient scores result from 10 different models (one from each cross validation fold) with 10 different score thresholds. The data set of origin is indicated for each sample at the bottom of each bar

First, rather than designate a single validation cohort, we repeatedly selected different validation cohorts to produce a statistically robust evaluation of performance that is independent from the selection of a single validation cohort. In particular, we used repeated 10-fold cross validation to provide a nearly unbiased assessment (slight underestimate) of classifier performance [25]. The classifier performed with high specificity (median specificity = 92 %, median precision = 60 %), while still correctly identifying a significant fraction of non-responders (median sensitivity = 31 %; Table 3). The median area under the receiver operating characteristic curve (AUROC) was 71 % (with a 95 % confidence interval (CI) of 60-81 %, p-value = 0.0003; Table 3, Fig. 1a). When repeated 10-fold cross validation was performed while randomizing the response calls for each repeat, a median AUROC of 50 % was achieved, and only eleven of the 1000 repeats produced AUROCs above 71 %, further confirming the significance of the classifier performance. The classifier had a median cross-validated likelihood ratio of 3.94, indicating that it was able to identify a subpopulation enriched in non-responders (~0.75:1 responder to non-responder ratio) compared to the full population (~2.5:1 responder to non-responder ratio), and place nearly one third of the non-responders in this subpopulation. Clinically, this would correspond to providing almost one third of the non-responders with a prediction that treatment with an alternative biologic could reduce their likelihood of non-response from 60 % (the test precision, corresponding to the 0.75:1 responder to non-responder ratio) to 30-40 % (the non-response rates of other biologics).

**Table 3:** Classifier performance assessed via repeated 10-fold cross validation

Data sets	Treatment	Patient breakdown	Median AUROC(95 % CI)	Median specificity(95 % CI)	Median sensitivity(95 % CI)	Median precision(95 % CI)	Median likelihood ratio(95 % CI)
GSE12051, GSE19821, GSE58795, GSE33377_infliximab_	Infliximab	32 NR, 84 R	71 %^**^(60-81 %)	92 %(84-97 %)	31 %(16-50 %)	60 %(36-81 %)	3.94(1.78-8.7)

Repeated 10-fold cross validation performance for a classifier trained on four whole blood infliximab data sets

*R*, responder; *NR*, non-responder; *CI*, confidence interval. ** indicates AUROC p-value < 0.01

Second, to evaluate the ability of the classifier to predict response in independent patient cohorts (i.e., in cohorts collected completely independently from any samples used in the training), leave-one-batch-out cross validation was performed, in which only three of the four whole blood data sets were used in training and the fourth left out to be used exclusively for testing [27]. This out-of-batch cross validation was repeated such that each data set served as the left-out test. This approach was preferable to designating a single data set at the test data set as it allowed us to assess the performance for each possible selection of a test data set, and allowed us to observe and average across the variability from one small test cohort to the next. Because this approach relied on training on fewer patients (between 72 and 101, depending on which data set is treated as the validation cohort), it provided an underestimate of the performance expected from training on the full set of 116 patient. With a mean AUROC of 71 %, the leave-one-batch-out performances were generally consistent with the 10-fold cross validation performance (Table 4): 82 % (p-value = 0.003; GSE12051), 90 % (p-value = 0.006; GSE19821), 57 % (p-value = 0.32; GSE58795), and 57 % (p-value = 0.28; GSE33377_infliximab_) (Fig. 1b). The smaller number of patients used in each test cohort led to significantly more variability and wider confidence intervals relative to the 10-fold cross validation (Table 4). Classification of non-responders in each left-out cohort was again similar to that observed with 10-fold cross validation, with a mean specificity of 94 %, a mean precision of 69 %, and a mean sensitivity of 32 % across all four data sets (Table 4). These results demonstrate that a classifier trained across multiple patient cohorts can predict response in an independent cohort with performance similar to in-batch cross validation. Furthermore, the significant variability observed in performance between data sets supports the use of leave-one-batch-out cross validation instead of designating a single data set as the test cohort.

**Table 4:** Classifier performance assessed via leave-one-batch-out cross validation

Left out data set	Treatment	Patient breakdown	AUROC(95 % CI)	Specificity(95 % CI)	Sensitivity(95 % CI)	Precision(95 % CI)	Likelihood ratio(95 % CI)
GSE12051	Infliximab	7 NR, 37 R	82 %^**^(66-98 %)	97 %(86-100 %)	14 %(0-58 %)	50 %(1-99 %)	5.29(0.4-74.9)
GSE19821	Infliximab	5 NR, 10 R	90 %^**^(74-100 %)	100 %(59-100 %)	40 %(5-85 %)	100 %(9-100 %)	∞(N/A)
GSE58795	Infliximab	7 NR, 23 R	57 %(26-87 %)	91 %(72-99 %)	43 %(10-82 %)	60 %(15-95 %)	4.93(1.0-23.8)
GSE33377	Infliximab	13 NR, 14 R	57 %(34-80 %)	86 %(57-98 %)	31 %(9-61 %)	67 %(22-96 %)	2.15(0.5-9.9)

Leave-one-batch-out cross validation performance for a classifier trained on three of the four whole blood infliximab data sets, and tested on the left-out data set. The likelihood ratio for GSE19821 is infinite and the confidence interval cannot be calculated because no responders were labeled as non-responders by the classifier

*R*, responder; *NR*, non-responder; *CI*, confidence interval. ^**^ indicates AUROC p-value < 0.01

### Classifier error due to uncertainty in DAS28 scores

The response calls in all of the whole blood infliximab data sets are based on DAS28 scores (Table 1), which are known to have a measurement standard deviation of 0.6 [26]. By simulating variability in DAS28 scoring, we found that even if the true disease activity were known, the variability in measuring DAS28 would limit the performance of a DAS28-based classifier to have an AUROC no greater than 89 % (see Methods). This analysis provides an upper limit on the ability of a classifier to differentiate between responders and non-responders in RA.

### Prediction consistency between whole blood and PBMCs

Because PBMCs are a sub-set of whole blood with the vast majority of disease relevant cells [28, 29], we sought to evaluate how well the classifier, which was trained on whole blood data, performed on two PBMC infliximab data sets: GSE42296 and GSE3592 (Table 1). The classifier provided reasonable stratification in one data set (GSE42296; AUROC = 74 %, p-value = 0.053) but not in the other (GSE3592; AUROC = 55 %, p-value = 0.42; Table 5), consistent with the performance of the classifier in leave-one-batch-out cross validation.

**Table 5:** Classifier performance on additional RA test cohorts

Data set	Treatment	Blood sample	Patient breakdown	AUROC(95 % CI)	Specificity(95 % CI)	Sensitivity(95 % CI)	Precision(95 % CI)	Likelihood ratio(95 % CI)
GSE42296	Infliximab	PBMCs	13 NR, 6 R	74 %(51-98 %)	100 %(42-100 %)	31 %(9-61 %)	100 %(28-100 %)	∞(N/A)
GSE3592	Infliximab	PBMCs	7 NR, 6 R	55 %(19-90 %)	67 %(22-96 %)	14 %(0-58 %)	33 %(1-91 %)	0.43(0.1-3.6)
GSE33377	Adalimumab	Whole blood	11 NR, 4 R	61 %(22-100 %)	100 %(28-100 %)	18 %(2-52 %)	100 %(9-100 %)	∞(N/A)
GSE11827	Anakinra	PBMCs	7 NR, 7 R	29 %(0-65 %)	100 %(47-100 %)	14 %(0-58 %)	100 %(1-100 %)	∞(N/A)
GSE25160	Tocilizumab	PBMCs	4 NR, 9 R	50 %(9-91 %)	100 %(47-100 %)	25 %(1-81 %)	25 %(1-81 %)	0.75(0.1-5.2)
GSE37107	Rituximab	Whole blood	6 NR, 8 R	33 %(0-72 %)	75 %(35-97 %)	33 %(4-78 %)	50 %(7-93 %)	1.33(0.3-6.9)

*R*, responder; *NR*, non-responder, *CI*, confidence interval. No AUROC p-values were less than 0.05

The successful stratification in one of the PBMCs data sets prompted investigation of whether it would be possible to train a classifier to predict response to infliximab treatment across both whole blood and PBMC. Repeated 10-fold cross validation of a classifier trained on all six infliximab data sets resulted in a median AUROC of 69 % (95 % CI = 60-78 %, p-value = 9 × 10^−5^), and leave-one-batch-out cross validation produced similar performance (mean AUROC = 69 %): 59 % (p-value = 0.23; GSE12051), 82 % (p-value = 0.03; GSE19821), 66 % (p-value = 0.11; GSE58795), 68 % (p-value = 0.06; GSE33377_infliximab_), 71 % (p-value = 0.09; GSE42296), and 67 % (p-value = 0.18; GSE3592). There was no apparent difference in performance between the whole blood and PBMC data sets. Together, these results suggest that there may be sufficient common signal across PBMCs and whole blood for the prediction of response to anti-TNF therapy.

### Comparison with previously published classifiers

**Fig. 2:**
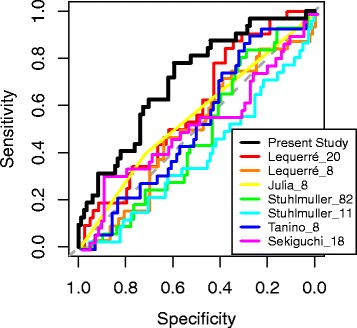
Comparison of classifier with previously published classifiers. The receiver operator characteristic (ROC) curves for our classifier and each previously published classifier show the sensitivity and specificity relationships for different thresholds. The ROC curve for our classifier results from a representative 10-fold cross validation repeat, matching Fig. 1. The ROC curves for the other classifiers result from leave-one-sample-out cross validation. The ROC curve for our classifier is the only one that demonstrates a strong consistent bias to the upper left, consistent with it being the only one with a statistically significant AUROC. Because Julia_8 only produces classifier scores of 0 or 1, many samples have the same score which leads to the non-stepwise behavior of the corresponding ROC curve. The dashed grey line indicates the null hypotheses of random stratification, corresponding to an AUROC of 50 %

A previous study evaluated the performance of eight published anti-TNF gene expression classifiers using a single dataset, GSE33377 [16]. We sought to compare these same eight classifiers, which were developed using either whole blood or PBMCs (see Methods), to that in the present study using the four whole blood infliximab data sets used here. One classifier (Stuhlmuller_3) was omitted because only one of the three genes in this classifier was measured across all four whole blood data sets. Using the selected genes and clustering method from each of these classifiers (see Methods), we performed leave-one-sample-out cross validation across the four whole blood data sets (Fig. 2). All classifiers produced AUROCs that were substantially less than the 71 % observed in the current study, and none were statistically significant (p-value < 0.05; Table 6): 59 % (p-value = 0.06; Lequerré_20), 50 % (p-value = 0.50; Lequerré_8), 57 % (p-value = 0.11; Julia_8), 48 % (p-value = 0.61; Stuhlmuller_82), 40 % (p-value = 0.94; Stuhlmuller_11), 54 % (p-value = 0.26; Tanino_8), and 53 % (p-value = 0.30; Sekiguchi_18). Although its performance was not statistically significant, Lequerré_20 was identified as the best performing of these classifiers here as well as in a previous study [16].

**Table 6:** Performance of previously published classifiers assessed via repeated leave-one-sample-out cross validation

Classifier	AUROC(95 % CI)	Specificity(95 % CI)	Sensitivity(95 % CI)	Precision(95 % CI)	Likelihood ratio(95 % CI)
Lequerré_20	59 %(48-70 %)	58 %(47-69 %)	50 %(32-68 %)	31 %(19-46 %)	1.2(0.78-1.8)
Lequerré_8	50 %(38-62 %)	61 %(50-71 %)	47 %(29-65 %)	31 %(19-46 %)	1.19(0.76-1.9)
Julia	57 %(48-69 %)	74 %(60-86 %)	40 %(21-61 %)	45 %(24-68 %)	1.57(0.79-3.1)
Stuhlmuller_82	48 %(37-59 %)	40 %(30-52 %)	63 %(44-79 %)	29 %(18-41 %)	1.05(0.76-1.4)
Stuhlmuller_11	40 %(29-52 %)	43 %(32-54 %)	47 %(29-65 %)	24 %(14-36 %)	0.82(0.54-1.2)
Tanino	54 %(43-65 %)	51 %(40-62 %)	50 %(32-68 %)	28 %(17-42 %)	1.02(0.68-1.5)
Sekiguchi	53 %(41-66 %)	24 %(15-34 %)	75 %(57-89 %)	27 %(18-38 %)	0.98(0.78-1.2)

Each classifier was re-trained on the four whole blood data sets (GSE12051, GSE19821, GSE58795, GSE33377_infliximab_) using the genes and clustering method specified in the original studies. The performance of each classifier was assessed via leave-one-sample-out cross validation across. Julia_8 was originally trained on GSE12051, so GSE12051 was omitted from cross validation for Julia_8 to avoid feature selection bias

CI: confidence interval. No AUROC p-value < 0.05

### Classifier prediction on other biologics

The strategy of using a classifier to direct some RA patients away from first-line biologic anti-TNF therapies would only prove effective if these patients were viable candidates to respond to second- and third-line biologic therapies. Thus, it was also important to evaluate whether patients identified by the classifier as likely non-responders to infliximab therapy tended to be non-responders to other biologics. We thus identified additional data sets where RA patients were treated with biologic therapies, and where the specific treatment and subsequent response call was unambiguously known for each patient (Table 1). When the performance of the present classifier was evaluated in RA cohorts treated with other targeted biologic therapies, it was observed that stratifications were not significantly associated with non-response to adalimumab, anakinra, tocilizumab, or rituximab (Table 5). Note that the anakinra and tocilizumab data sets were PBMC samples, and thus interpretation of these results requires the additional assumption that the classifier can be applied across blood sample types. However, the limited size of all abovementioned cohorts and the use of PBMCs in two of these studies does not preclude the possibility that statistically significant associations between the classifier score and patient response to other therapies may be found in larger cohorts.

### Biological characterization of Infliximab Non-responders

**Fig. 3:**
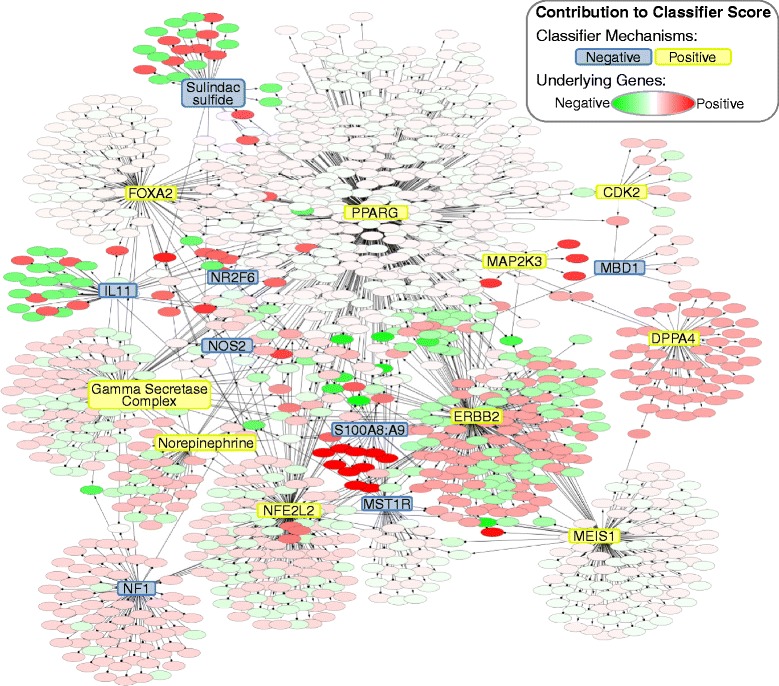
Contributions of classifier mechanisms and underlying genes to classifier score. Each classifier mechanism is connected to the genes used to compute its score. Mechanisms are colored based on their direction of contribution to the classifier score (the sign of the coefficient from Table 2). Genes are colored based on their direction and degree of contribution to the classifier score, calculated from their contribution to each mechanism score and the coefficient for each mechanism. Interconnectedness indicates the overlaps between the gene sets that contribute to each mechanism score. Mechanisms with large numbers of supporting genes tend to have lower contributions at the gene level due to the equal contribution of each supporting gene to the mechanism score (see Methods), effectively diluting the contribution of each gene. Gene names were omitted for clarity. See Additional file 3 for the full Cytoscape model

The 18 mechanisms in the classifier are known to regulate numerous biological pathways, most predominately response to wounding (FOXA2, ERBB2, IL11, MAP2K3, NF1, S100A8:S100A9), immune defense response (gamma secretase complex, IL11, MAP2K3, MST1R, NOS2, NR2F6, PPARG, S100A8:S100A9, sulindac sulfide) and nervous system development (FOXA2, gamma secretase complex, MEIS1, NF1, norepinephrine, PPARG). Noted exceptions are DPPA4 and MBD1, for which their functions are not well understood, and CDK2, which functions ubiquitously in tissues undergoing proliferation. The 18 mechanism scores were computed from between 1110 and 1387 total unique genes, depending on the microarray platform used in each study (see Additional files 2 and 3), with significant overlaps between the gene sets for each mechanism (Fig. 3). These genes were further analyzed for significant enrichment of functional annotations using the ToppGene bioinformatics resources [30] (Additional file 4). Increased expression of genes involved in defense, wounding, and inflammatory responses supported classifier prediction of response. The signaling pathways most enriched for genes with increased expression supporting a response prediction were TNF, NF-κB, and HIF1A. In contrast, non-response prediction was supported by increased expression of genes involved lipid and drug metabolism pathways, and specifically fatty acid oxidation in response to hormone stimuli.

## Discussion

### Clinical feasibility of predicting anti-TNF Non-response

As the current treatment paradigm largely uses anti-TNF therapy after the failure of oral disease-modifying antirheumatic drug (DMARDs), we selected the classifier to minimize the false identification of non-responders (i.e., the incorrect identification of responders as potential non-responders) and maximize the specificity of the classifier. A classifier prediction of non-response would inform the physician that the patient has an elevated likelihood of failure to anti-TNF therapy, and treatment of this patient with an alternative biologic therapy may reduce their likelihood of non-response by a third to a half (based on the classifier’s likelihood ratio of 3.75, and the overall likelihood of non-response of 30-40 % for anti-TNFs and other biologics [7]). Such a strategy that increases the likelihood of response could more rapidly control disease progression, significantly affect quality of life, and save tens of thousands of dollars in ancillary healthcare costs associated with each ineffectively-treated patient [31].

While the reported performance of the classifier could indeed provide clinical utility, largely due to the equal effectiveness of alternative biologics compared to anti-TNF therapy, additional data is required to validate the classifier. In addition to validating the current classifier, additional data sets could be used to further refine the classifier. The imperfect classification offered by the classifier can be attributed at least in part to experimentally controllable factors such as the differences in sample collection and processing protocols, microarray platform, and patient clinical characteristics. Thus, it is reasonable to expect increased performance in future iterations of the classifier based on fully-consistent training data. It remains unclear how close classifier performance could get to the maximum 89 % AUROC performance expected due to DAS28 measurement error.

The validity of the classifier is further supported by the relevance of the classifier mechanisms and their underlying genes to RA. The mechanisms and underlying genes of the classifier suggested that the blood of anti-TNF non-responders may generally have lower cytokine signaling involved in inflammation, including TNF and NF-κB pathways, while having higher levels of specific metabolic activities associated lipolysis and fatty acid oxidation, compared to responders. Systemic inflammation, and TNF specifically, have been suggested to influence the metabolic dysregulation, atherosclerosis, and higher risk for cardiovascular disease associated with RA [32, 33]. Although blocking TNF activity has been demonstrated to reverse some of the effects of the metabolic syndrome observed in RA [34, 35], the specific mechanisms in the classifier have not, to our knowledge, previously been associated with response to anti-TNF treatments. Future studies may elucidate specific roles for these mechanisms in the response to anti-TNF therapy, or may potentially reveal that downstream genes regulated by the mechanisms in the classifier overlap with others more directly linked to TNF-related pathways.

### Limitations of the work

The main limitations of this work involve the relatively limited number of samples used for classifier training and evaluation. The variability in performance in the leave-one-batch-out analysis may result from the failure of the classifier to predict in some cohorts, or may simply result from the small number of samples in each data set (suggested by the same average performance observed in repeated 10-fold cross validation). While the use of retrospective samples in various internal validation cohorts (through repeated 10-fold and leave-one-batch-out cross validation) is statistically valid and rigorous, ultimately a large prospective study is needed for classifier validation. Furthermore, a well-controlled prospective study would enable collection of appropriate clinical information, which was generally missing for individual patients from the published data sets used herein. Evaluation or refinement of the classifier on a larger number of patients would provide more precise estimates of performance (smaller confidence intervals), and would be needed to provide physicians with sufficient evidence that such a test could indeed be used to help guide patient treatment.

## Conclusions

The results of the present study demonstrate that by training across data from multiple studies we were able to produce a predictive classifier for non-response to anti-TNF treatment that is robust to the test population. Given significant differences in batch, microarray platform, and patient populations, this initial effort integrating distinct experimental data sets suggests that a classifier to identify non-responders is achievable with the current methodology. Additional data is required to prospectively validate the classifier and provide additional refinement.

## Additional files

Additional file 1:
**Mechanism strength scores.** Mechanism strength scores for all 1667 mechanisms for the training data sets, and for the 18 classifier mechanisms for all data sets presented herein. Additional file 2:
**Classifier mechanisms.** This file contains the genes used to compute the strength score for each of the 18 mechanisms in the classifier, as well as the direction of contribution of each gene to each mechanism score. Additional file 3:
**Classifier mechanisms.** This file is a cytoscape model of the same information presented in Additional file 1, and used to generate Fig. 3. Additional file 4:
**Functional enrichment analysis of classifier gene functions.** Genes in the classifier were divided into those that contributed positively to the score (supporting prediction of anti-TNF non-response) and those that contributed negatively to the score (supporting prediction of anti-TNF response). ToppGene was used to detect enrichment within each gene list based on functional ontologies (GO Biological Process, pathways) and phenotype [30]. Results were filtered to provide the lowest 25 by false discovery rate adjusted p-value (FDR B&H). WikiPathways were excluded from the analysis due to the non-canonical or speculative nature of several pathways with significant enrichment scores.
